# A Narrative Review of Function-Focused Measures for Children With Neurodevelopmental Disorders

**DOI:** 10.3389/fresc.2021.709978

**Published:** 2021-07-29

**Authors:** Kajaani Shanmugarajah, Peter Rosenbaum, Mohammad Zubairi, Briano Di Rezze

**Affiliations:** ^1^School of Rehabilitation Sciences, McMaster University, Hamilton, ON, Canada; ^2^CanChild Centre for Childhood Disability Research, Hamilton, ON, Canada; ^3^Department of Pediatrics, McMaster University, Hamilton, ON, Canada; ^4^McMaster Autism Research Team, Hamilton, ON, Canada

**Keywords:** children, neurodevelopmental disabilities, clinical measures, ICF, NDD

## Abstract

Clinical measures in health and rehabilitation settings are often used to examine child functioning to better support the diverse needs of children with neurodevelopmental disorders (NDD) and their families. The WHO's International Classification of Functioning, Disability, and Health (ICF) framework reflects a focus of health beyond biomedical deficits, using the concept of functioning to create opportunities for measurement development involving this construct. In the measures developed in the field of childhood NDD, it is unclear whether and how these tools measure and incorporate the ICF framework and its domains within health care contexts. Understanding how these measures utilize the ICF will enable researchers and clinicians to operationalize function-focused concepts in studies and clinical practice more effectively. This narrative review aims to identify and describe function-focused measures that are based on the ICF for children with NDD, as described in the peer-reviewed literature. This review used a systematic search strategy with multiple health-focused databases (Medline, PsycInfo, EMBASE, EMCARE), and identified 14 clinical measures that provide direct support for children (aged 0-21) with NDD in pediatric health (and other) settings. Results described the measures that were primarily developed for three main *diagnostic populations* [cerebral palsy, autism spectrum disorder, and communication disorders]; had varying *contextual* use (clinical-only or multiple settings); and for which authors had conducted *psychometric tests* in the measure's initial development studies, with the most common being content validity, interrater reliability, test-retest reliability. Participation (79%, *n* = 11) & Activities (71%, *n* = 10) were the most common ICF domains captured by the set of measurement tools. Overall (71%, *n* = 10) of the identified measures utilized multiple ICF domains, indicating that the “dynamic nature” of the interactions of the ICF domains was generally evident, and that this result differentiated from “linking rules,” commonly used in research and clinical practice. The implications of these findings suggest that clinical measures can be an effective application of the ICF's defined concepts of functioning for children with NDD.

## Introduction

Neurodevelopmental disorders (NDD), as defined by the Diagnostic and Statistical Manual of Mental Disorders, 5th Edition (DSM-5), refer to a group of conditions that present during a child's early developmental period and are characterized by developmental deficits that may create challenges in the child's personal, social, academic, or occupational functioning (1). Common examples of NDD include autism spectrum disorder (ASD), communication and/or language disorders, attention deficit/hyperactivity disorder, motor disorders (including cerebral palsy [CP]), learning disorders, and developmental coordination disorder (1). The prevalence rate for NDD in developed countries range from 7 to 14% of all children (2). Children with NDD may experience challenges in different environments, potentially impacting their functioning within academic settings (school), daily living skills (home), and the broader community (3–7). The DSM-5 describes these challenges as a symptom of excess, deficit, or delay in key aspects of child functioning, especially when considering the achievement of expected developmental milestones (1).

Historically, biomedical models and thinking have greatly influenced clinical practice, including the field of childhood disability (8, 9). This traditional way of thinking focused on the attributes of a child's deficits and limitations, for diagnostic purposes and to treat aspects of the child's “disability” (10, 11). In 2001, the International Classification of Functioning, Disability, and Health (ICF) – a contemporary conceptual framework – challenged these practices and highlighted the paradigm shift to think beyond the biomedical model to an integrated biopsychosocial model of human functioning and disability (8). This biopsychosocial model emphasizes that individuals with disabilities have needs that extend beyond the medical scope of practice, and are often broad-based in nature within social, educational, and functional settings (12).

**Figure 1:**
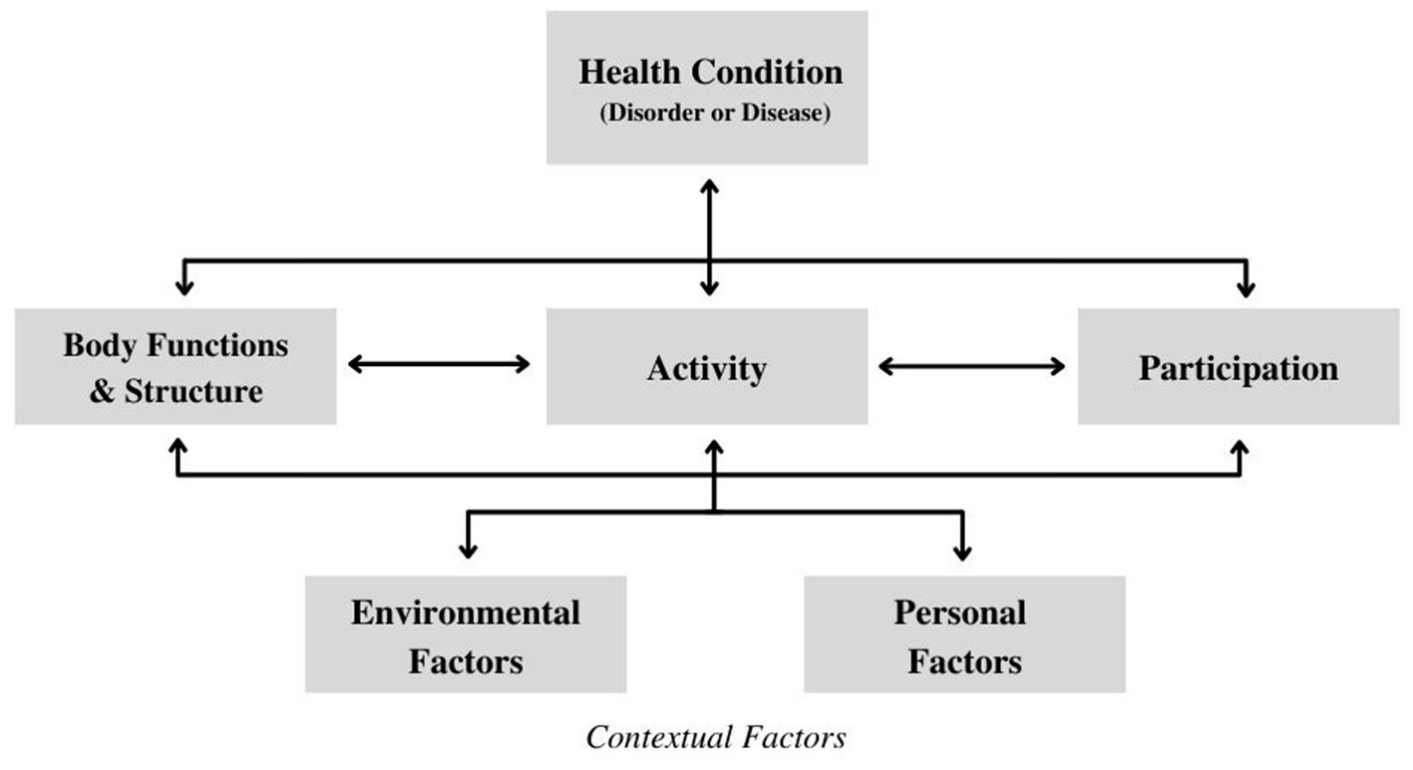
International classification of functioning, disability, and health framework (8).

As shown in Figure 1, the four key domains of the ICF are: *body function* & *structures* (functioning at the level of the body); *activities* (functioning at the level of the individual); *participation* (functioning of a person as a member of society); and *contextual factors* (personal and environmental factors that can exist as facilitators or barriers) (8). The ICF framework defines *functioning* as an umbrella term to describe the interactions of these four domains, examining the positive or neutral aspects occurring between the individual's health condition(s) and their context (8). “Disability” is an alternate umbrella term used to describe the interactions of an individual's impairments, activity limitations and participation restrictions, examining the negative aspects of the *interaction* between the individual's health condition(s) and their context (8).

This ICF framework depicts the interactive and non-linear nature of the core domains, establishing that these conceptual domains are not independent when examining functioning and/or disability. The framework is representative of the biopsychosocial perspective, as it recognizes how the influences of physical, psychological, and social factors within “functioning and disability” can be understood from the viewpoint of the individual with respect to their health condition (13, 14). Without focusing on single descriptors to label a child's functional abilities, this framework utilizes a holistic approach that still highlight the nuances in the interactions of the different elements that build a child's functional profile (14, 15). This framework indicates a paradigm shift in the ways that researchers and clinicians understand disability, as it provides a multidimensional perspective that both classifies functioning independently from the individual's diagnosis and views disability as product of person-environment interactions (12, 15, 16).

Children with NDD exhibit a wide range of levels of functioning within and between their diagnostic groups (17). In addition to the ICF framework, the field of NDD has seen growth in the concept of functioning that takes into consideration the heterogeneous level of abilities within diagnostic populations that extends across NDD (10). For example, within ASD literature, the concept of *neurodiversity* views neurological differences as inherent human variation, rather than as a disorder, and celebrates the individuality of a person – regardless of their capabilities (18, 19). This change in thinking in the field of ASD has had a great influence in promoting various abilities and child differences within ASD interventions, including the language that is being used to describe the diagnosis (19, 20).

Similar concepts of functioning started in the field of CP, in relation to interventions in pediatric rehabilitation. Rather than using the traditional approaches of CP that attempt to normalize movement patterns and minimize the development of secondary impairments, there is an increased emphasis on enabling the child to master various tasks and participate in different activities (21, 22). Over the last 20 years, in the field of CP, clinical care and research have examined child functioning as it relates to interacting contextual factors (22, 23). Although the needs and abilities of children with NDD (and their families) are heterogeneous, everyday functioning is continually regarded as an important outcome to families (24).

Examining clinical contexts in particular, there is a notable emphasis of functioning in the ICF, as this term is often used to describe abilities-focused processes – otherwise commonly referred to as function-focused care (12, 24, 25). Within this type of pediatric care planning, there are typically certain measures used with families to promote child functioning or child abilities.

Although these measures may have the appropriate psychometric testing completed to illustrate their effectiveness in clinical utility, it is also important that there is some consistency with the language that is being used with these measures (20, 26, 27). For example, the terms “function,” “functional,” and “everyday functioning,” are used synonymously in the literature, whereas the ICF's conceptualization of functioning emphasizes it as the complex interactions between the four domains (10). There are various measures that aim to assess concepts related to function, such as adaptive behavior [e.g., Vineland Adaptive Behavior Scales (28), Adaptive Behavior Assessment System (29), Behavior Assessment System for Children (30)]. However, these measures are not based on the ICF, and therefore describe everyday function differently compared to ICF-based measures.

Operationalizing the ICF framework (i.e., its domains and the interactions between them) within measurement tools can create opportunities for the ICF to be widely utilized in clinical environments for children with NDD (14). It is unclear how many measures in the field of NDD are developed using the definitions and concepts of the ICF framework. It has been demonstrated in the literature that clinical measures can be mapped on or “linked” to the ICF framework by following a set of established and standardized rules, as described by Fayed et al. (31). With NDD interventions shifting to focus more specifically on strengths and support needs, there is a need for further description of how measures that purport to be function-focused are utilized in clinical systems. The focus of this study is directed toward examining how researchers who have developed ICF-based measures conceptualized their measure, specifically with whether and how the ICF domains were utilized in pediatric clinical contexts and research.

## Methods

We undertook a narrative review and synthesis of the peer-reviewed literature to understand existing function-focused, ICF-based measures that are used with children diagnosed with NDD. A narrative review summarizes and describes previously published information with an interpretation of the contents of different studies using a comprehensive, critical and objective analysis (32, 33). This study was guided by SANRA, the Scale for the Assessment of Narrative Review Articles, specifically by reviewing the six items deemed necessary for a quality review: (1) justification of the review's importance for the reader; (2) review focus/aim(s); (3) description of literature search; (4) referencing; (5) scientific reasoning; and (6) relevant and appropriate endpoint/presentation data (33).

We used a two-stage approach to review the literature. The first stage was to identify original research texts that (a) focused on children (18 and younger) diagnosed with NDD defined by DSM-5, and (b) referenced the ICF framework. Both criteria needed to be stated within the abstract of the study. Initial keywords were generated for each conceptual category of the research aim with the guidance of a trained librarian in a health sciences library to form the search strategy. Keywords were identified within three categories (NDD diagnosis, child [age range 0–18], and ICF), and were used to search the following databases: Medline, PsycInfo, EMBASE, and EMCARE. Search terms were developed and customized for each database. Abstracts were then screened to identify whether any measures were used in an intervention study and/or discussed in the literature; we also required the clinical measure to be the focus of the abstract. Searches were restricted to both English language journals and publication date (2002-April 2021), as selected papers were required to be published post-publication of the ICF in 2001. Studies were excluded if the aim was to translate the measure to explore psychometric properties within an alternate language/country/context. Measures that were used within indirect care (i.e., measures that focused on data collection and/or inter-professional collaboration) were also excluded. Lastly, secondary studies (i.e., systematic reviews, scoping reviews) as well as editorials and commentaries were excluded. The titles and abstracts of the resulting articles from the database search were exported to Covidence (34), a reference managing software. Duplicate records were then deleted using the software.

The second stage required full-text screening to identify whether select measures were ICF-based, and to identify whether the study reported the development of the measure. If a study described an ICF-based measure but was not the original paper of the measure's development, hand-searching was conducted to retrieve the original article describing its development. Hand-searching for original articles was accomplished by looking at the reference lists of the indexed articles that had described the use of these measures within their abstracts. This task was also completed using Covidence (34).

This study used a matrix to extract key details including age ranges, context(s), diagnosis sample, as well as descriptions and psychometric properties of the measures described. Details and descriptions of these measures were determined by using its original development article. After the characteristics of the included measures were extracted, the original studies of the measures were analyzed again to extract ICF-related details, specifically regarding the ICF domain(s) that were prioritized by the measure and how this framework influenced the measure's initial conception. Measures were categorized by using the definitions of the four domains of the ICF framework (body structures and function, activities, participation, and contextual factors).

## Results

The initial search identified 2811 published abstracts. After duplicates were removed, 1947 papers remained. These papers were reviewed by title and abstract with the first set of inclusion criteria, resulting in 141 potentially relevant studies. For the second stage, full-text versions of these studies were obtained and reviewed to assess whether they fit the second set of inclusion criteria, at which time 97 studies were excluded. Studies were excluded mainly for having a non-relevant focus—not focused on an ICF-based measure providing direct support for children with NDD; focusing on a non-pediatric sample; or being based on secondary data. The 44 papers that remained included 9 studies that described the initial development of an ICF-based measure, and 35 articles that described the use of ICF-based measures but were not the measure's original development paper, for which hand-searching was then necessary.

**Figure 2:**
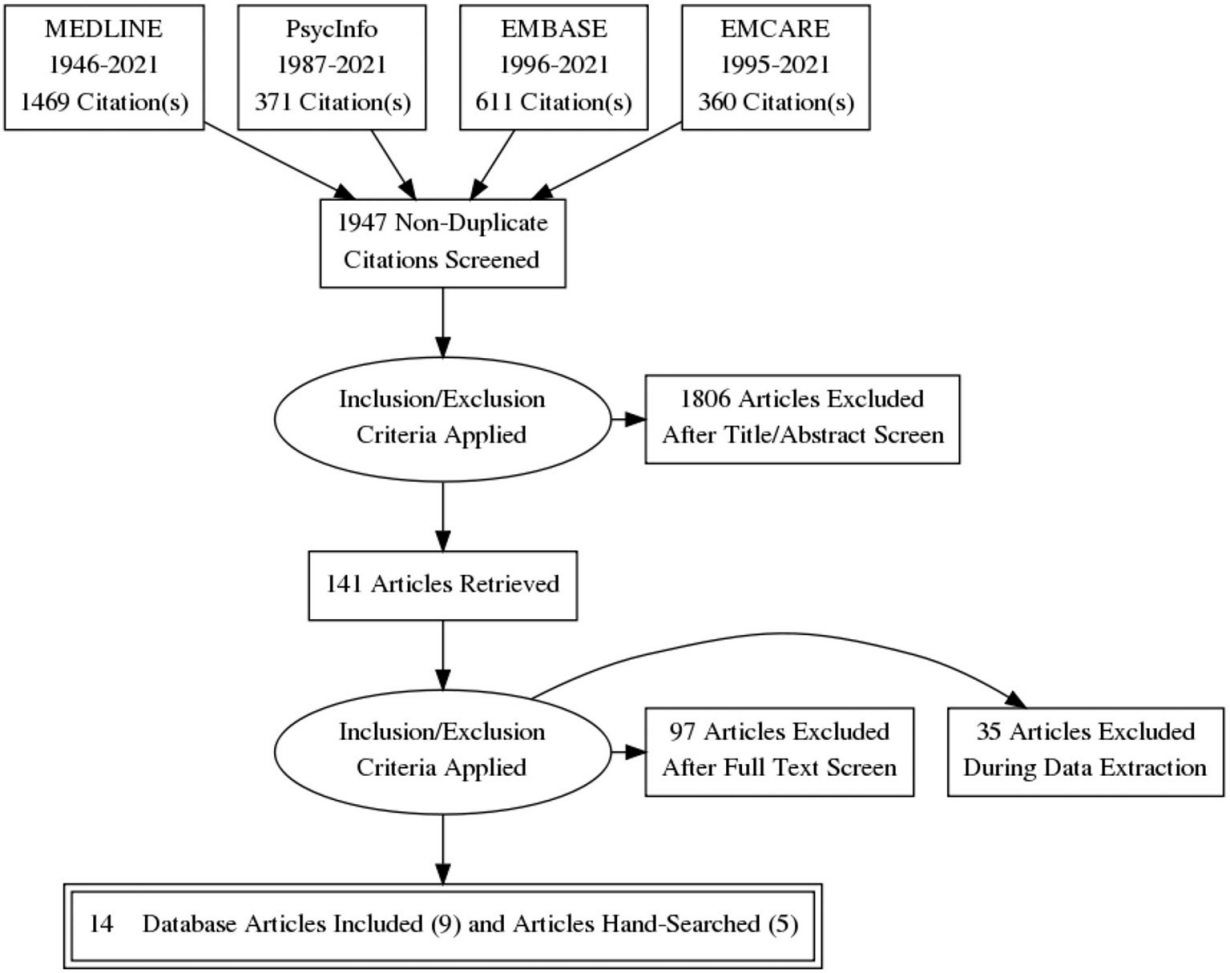
PRISMA (40) flowchart of search strategy.

From the 35 articles, five additional ICF-based measures for children with NDD were identified and included; these comprised of four additional studies (35–38), and one manual (39). In total, 14 initial development studies describing 14 individual measures were included. This information is summarized using the Preferred Reporting Items for Systematic Reviews and Meta-Analyses (PRISMA) flowchart in Figure 2 (40).

The 14 measures originated in seven countries including Canada, US, UK, Australia, Sweden, Switzerland, and Taiwan. These measures were predominately described as either assessment and/or outcome measures (64%, *n* = 9) or classification systems (36%, *n* = 5), and could be utilized in various contexts including home, community, educational, and clinical environments. The most common diagnosis was CP (50%, *n* = 7), followed by non-diagnostic/multiple diagnoses (29%, *n* = 4), ASD (14%, *n* = 2), and communication disorders (7%, *n* = 1). Age applicability of these measures ranged from 0 to 21 years. The diagnosis sample, age groups, and brief descriptions are reported in Table 1. The common characteristics of these measures are also described.

**Table 1:** General description and characteristics of ICF-based clinical measures.

**Measure acronym**	**Measure full name and citation**	**Type of measure**	**Country of origin**	**Primary context(s)**	**Diagnosis sample**	**Age range**	**Construct of interest**	**Brief description**
ICF-CS	ICF Core Set (41)	Standardization for Assessment and Description	Switzerland	Multiple (i.e., clinical, home, educational, community)	Multiple versions with different diagnoses (ASD, ADHD, CP)	Multiple versions with different age ranges	Functional Abilities	This measure uses select categories from the ICF classification to describe relevant information in regards to an individual's level of functioning; this helps facilitate a systematic and comprehensive system for either a specific health condition or health context (41). There are two versions of this measure: ICF Comprehensive Core Sets and ICF Brief Core Sets (41).
GMFCS – E&R	Gross Motor Function Classification System Expanded and Revised (35)	Classification System	Canada	Clinical	Cerebral Palsy	0–18 years	Gross Motor Function	A 5-level classification system that describes gross motor function for children and youth with CP, specifically focused on self-initiated movement when a child sits, walks, and/or uses a wheeled mobility device (35).
MACS	Manual Ability Classification System (42)	Classification System	Sweden	Clinical	Cerebral Palsy	4–18 years	Manual Ability	Developed from the GMFCS, this 5-level classification system examines typical manual performance of children with CP, specifically in regards to a child's ability to handle objects (i.e., assistance needs, potential adaptations required, quantity/quality of performance) (42).
BFMF	Bimanual Fine Motor Function (43)	Classification System	Sweden	Clinical	Cerebral Palsy	Not specified	Fine Motor Function	A 5-level classification system that examines fine motor function in children with CP, specifically in regards to a child's ability to grasp, manipulate, and hold objects for each hand (43).
CFCS	Communication Function Classification System (44)	Classification System	USA	Multiple (i.e., clinical, home, educational, community)	Cerebral Palsy	2–18 years	Communication	A 5-level classification system used by clinicians for children with CP, to classify and understand the patterns of a child's performance in everyday communication effectiveness with a partner (44).
ACSF:SC	Autism Classification System of Functioning: Social Communication (36)	Classification System	Canada	Multiple (i.e., clinical, home, educational, community)	ASD	3–5 years	Social Communication	A 5-level classification system that provides a simplified method to describe social communication functioning for preschool children with ASD (36). This measure provides parents and service providers with an understanding of the potential differences in social communication abilities based on a child's capacity and typical performance within different contexts (36).
GOAL	Gait Outcomes Assessment List (45)	Assessment Measure	Canada	Clinical	Cerebral Palsy	Not specified	Gait Priorities	An assessment measure that evaluates gait priorities and functional mobility for ambulant children with CP, addressing the spectrum of needs and/or goals of these children and their caregivers (45).
FOCUS^®^	Focus on the Outcomes of Communication Under Six (46)	Outcome Measure	Canada	Clinical	Communication Disorders	0–6 years	Communication	An outcome measure that evaluates change in communicative-participation, examining ‘real world' changes in preschool children's communication abilities (46).
QYPP	Questionnaire of Young People's Participation (47)	Assessment Measure	United Kingdom	Clinical	Cerebral Palsy	13–21 years	Participation	A 45-item questionnaire assessing participation frequency across multiple domains for children and adolescents with cerebral palsy (47).
MEVU	Measure of Early Vision Use (48)	Assessment Measure	Australia	Clinical	Cerebral Palsy	Not specified	Vision	A measure that examines typical performance with ‘how vision is used' during a child's everyday activities, interactions and environments (48).
CAP-HAND	Children's Assessment of Participation with Hands (37)	Assessment Measure	Australia	Multiple (i.e., clinical, home, educational, community)	No specific diagnosis	2–12 years	Participation	A parent report questionnaire, examining upper limb abilities across disorders, as well as the extent to which children participate in life situations (with a focus on hand use) (37).
CAPE & PAC	Children's Assessment of Participation and Enjoyment and Preferences for Activities of Children (39)	Assessment & Outcome Measure	Canada	Multiple (i.e., clinical, home, educational, community)	No specific diagnosis	6–21 years	Participation and Activity Preferences	Together, CAPE & PAC are self-report measures that examine children's participation and activity preferences within six dimensions of activity (39). CAPE documents the extent to which children with or without disabilities participate in everyday activities outside of their mandated school activities. PAC examines children's specific activity preferences (39).
-	ICF-CY Based Questionnaire (49)	Assessment Measure	Taiwan	Clinical	ASD	3–6 years	Functional Profile	This measures comprises of 118 items using the ICF-CY structure to evaluate the functional profiles of preschool children with ASD (49).
PEM-CY	Participation and Environment Measure for Children and Youth (38)	Outcome Measure	USA	Multiple (i.e., clinical, home, educational, community)	No specific diagnosis	5–17 years	Participation and Environment	This parent-reporting survey allows parents, researchers, and service providers to better understand a child's participation patterns in home, school, and community settings, by studying both participation and environmental factors at the same time (38).

### Assessment (and Outcome) Measures

The following nine ICF-based measures have a primary focus on the assessment of a specific construct of interest: ICF Core Sets (ICF-CS) (41), Gait Outcomes Assessment List (GOAL) (45), Focus on the Outcomes of Communication Under Six (FOCUS^®^) (46), Questionnaire of Young People's Participation (QYPP) (47), Measure of Early Vision Use (MEVU) (48), Children's Assessment of Participation with Hands (CAP-HAND) (37), Children's Assessment of Participation and Enjoyment & Preferences for Activities of Children (CAPE & PAC) (39), the ICF-CY Based Questionnaire (49), and the Participation and Environment Measure for Children and Youth (PEM-CY) (38). These types of measures describe details of functioning, can observe and evaluate a child's abilities and limitations within the construct of interest (otherwise referred to as outcome measures—a subset of assessment measures), and in some cases, it may be used to predict within-person change over time (26). These assessments can be completed by various individuals that are familiar with and/or are knowledgeable about the child's competencies within their daily routines, including caregivers, clinicians, and teachers (39). With the conceptual grounding of the ICF, these measures can provide a comprehensive and clinically useful understanding of a specific phenomenon, which can then be used for various applications within research and practice (39).

### Classification Systems

The remaining five ICF-based measures are classification systems that can be used for children with NDD: Gross Motor Function Classification System Expanded & Revised (GMFCS-ER) (35), Manual Ability Classification System (MACS) (42), Bimanual Fine Motor Function (BFMF) (43), Communication Function Classification System (CFCS) (44), and Autism Classification System of Functioning: Social Communication (ACSF:SC) (36). The GMFCS-ER (35), MACS (42), BFMF (43), and CFCS (44) each individually describe functioning in children with CP based on specific constructs (i.e., gross motor function, manual ability, fine motor function, and communication), and the ACSF:SC (36) describes social communication functioning in children diagnosed with ASD. In these classification systems, level I typically describes child functioning with the highest level of ability in that aspect of functioning, whereas levels IV-V typically describe child functioning with more significant limitations (43). The five levels in these systems are ordinal, describing different levels of a child's abilities for a specific construct (36). It is important to note that the differences between these levels are not equal, as these systems provide a simplified guide for families and clinicians to communicate level of functioning within the clinical process (43).

### Psychometric Properties of Development Studies

The studies in which these measures were first established were published between 2002 and 2021. Almost all measures were initially published in journal articles (93%, *n* = 13), with one measure [CAPE & PAC (39)] described in a manual format. Most studies of these measures (64%, *n* = 9) provided some psychometric testing information during the measure's development. The most common forms of testing include content validity, interrater reliability, and test-retest reliability (29%, *n* = 4). Other types of psychometric testing include various types of construct validity testing, such as discriminant validity, expert validity, and concurrent validity (each 7%, *n* = 1) or were generally described as construct validity (14%, *n* = 2). The CAPE & PAC (39). Manual did not specify the type of reliability and validity results (see Table 2).

**Table 2:** Psychometric properties described in the measures' initial development.

**Measure acronym**	**Development study**	**Diagnosis sample**	**Description of psychometric properties in development article**
ICF-CS	A guide on how to develop an International Classification of Functioning, Disability and Health Core Set (41)	Multiple versions with different diagnoses (ASD, ADHD, CP)	Not included in the development article.
GMFCS – E&R	Development of the Gross Motor Function Classification System for cerebral palsy (35)	Cerebral Palsy	Not included in the development article.
MACS	The Manual Ability Classification System (MACS) for children with cerebral palsy: scale development and evidence of validity and reliability (42)	Cerebral Palsy	External construct validation process was initiated, involving rehab professionals within pediatric rehabilitation and parents of children with CP (42). Interrater reliability was conducted using testing between parents and therapists (42).
BFMF	Neuroimpairments, activity limitations, and participation restrictions in children with cerebral palsy (43)	Cerebral Palsy	Not included in the development article.
CFCS	Developing and validating the Communication Function Classification System for individuals with cerebral palsy (44)	Cerebral Palsy	The second and third phases of the measure's development focused on revision and validation using nominal group studies and Delphi surveys (content validity) (44). The fourth phase measured interrater reliability among clinicians and parents as well as test-retest reliability (44).
ACSF:SC	Developing a classification system of social communication functioning of preschool children with autism spectrum disorder (36)	ASD	Interrater reliability reported good for parents and very good for professionals (36). Content validity of level descriptions and ratings were trialed by participants in each stage of measure development using surveys (36).
GOAL	The Gait Outcomes Assessment List: validation of a new assessment of gait function for children with cerebral palsy (45)	Cerebral Palsy	Concurrent validity was assessed comparing the GOAL with two related valid and reliable assessments of motor function (45). Further studies will be required with larger cohorts to assess validity and reliability of the GOAL in different populations (45).
FOCUS^®^	Development of the FOCUS (Focus on the Outcomes of Communication Under Six), a communication outcome measure for preschool children (46)	Communication Disorders	Parents completed the Pediatric Quality of Life Inventory (PedsQL) at the start and completion of treatment to evaluate FOCUS' content validity (46). Parents and clinicians completed the FOCUS measure twice within a 1 week period for test-retest reliability (46).
QYPP	The Questionnaire of Young People's Participation (QYPP): a new measure of participation frequency for disabled young people (47)	Cerebral Palsy	Test-retest reliability was examined by intra-class correlations using a two-way mixed model; results were comparable with other participation measures (i.e., GMFCS, MACS) (47). Using a rigourous expert review of the measure's item pool, content validity was maximized; known-groups (discriminant) validity was also supported (47).
MEVU	Measure of Early Vision Use: development of a new assessment tool for children with cerebral palsy (48)	Cerebral Palsy	Not included in the development article.
CAP-HAND	Development and Psychometric Evaluation of a New Measure for Children's Participation in Hand-Use Life Situations (37)	No specific diagnosis	Evidence for construct validity was established using Rasch analysis. Differences in summary scores of each domain between children with and without disabilities were also significant (37). Test-retest reliability using ICCs of the measure was moderate-high, except for a single dimension scale. Internal consistency varied across the dimensions, providing preliminary evidence for construct validity and reliability (37).
CAPE & PAC	Children's Assessment of Participation and Enjoyment and Preferences for Activities of Children Manual (39)	No specific diagnosis	Information from the measure's longitudinal study was used to examine the technical characteristics of the CAPE and PAC (39). The data provided evidence of reliability and validity of the CAPE and PAC (39).
ICF-CY Based Questionnaire	ICF-CY based assessment tool for children with autism (49)	ASD	This measure has evidence of good interrater reliability, expert (construct) validity, and reflects the functional profile of preschool children with autism (49). Further testing is required to confirm other psychometric characteristics (49).
PEM-CY	Development of the participation and environment measure for children and youth: conceptual basis (38)	No specific diagnosis	Not included in the development article.

### ICF Domains of Measures

To understand the role of the ICF framework in the conception of these clinical measures, it was important to analyze what ICF domain(s) were prioritized, and the specific foundational concepts from the ICF framework during the initial development process (see Table 3). All listed measures included at least one domain of interest, and the ICF-CS (41), GOAL (45), and ICF-CY Based Questionnaire (49) using all four ICF domains. The most common domain across measures was Participation (79%, *n* = 11), followed by Activities (71%, *n* = 10), Contextual Factors (43%, *n* = 6), and Body Structures and Function (29%, *n* = 4). Seventy one percent of the measures (*n* = 10) used more than one domain of the ICF.

**Table 3:** ICF domains prioritized in the development of the measure.

		**ICF domains**			**Total domains**	**How is the ICF described overall?**
	**Body structures and function**	**Activities**	**Participation**	**Contextual factors**		
ICF-CS	✓	✓	✓	✓	4	This instrument selects essential categories that cover each component of the ICF.
GMFCS – E&R		✓		✓	2	“Our group's perspectives have evolved and been shaped considerably by the World Health Organization's (WHO) International Classification of Functioning, Disability and Health (ICF) […] The basic ideas concerning capacity and performance were included in the original GMFCS concepts but have been sharpened considerably with the publication of the ICF” [35, p. 251].
MACS		✓			1	“The focus is on manual ability, as defined in the International Classification of Functioning, Disability and Health [.] the classification looks at activities and gives a single ‘level' for the collaborative use of both hands when handling objects in daily life” [42, pp549-52].
BFMF	✓	✓	✓		3	“Motor function and learning disability were important predictors for participation restrictions in children with CP. The ICF has the capacity to be a model to help plan interventions for specific functional goals and to ascertain the child's participation in society” [43, p. 309].
CFCS		✓	✓		2	“The purpose of this study was to create and validate the Communication Function Classification System (CFCS) for children with CP, for use by a wide variety of individuals interested in CP. This required a shift from the traditional focus on body structure and function (i.e., assessing components of speech, language, and hearing problems), to a focus on activity/ participation, specifically the way in which to classify a person's communication capacity within real-life situations” [44, p. 705].
ACSF:SC		✓	✓		2	“Using the ICF activities and participation framework, resulting autism classifications will focus on how children's differing social communication affects their activities and participation in daily lives” [36, p. 943].
GOAL	✓	✓	✓	✓	4	“Used with gait analysis, the GOAL provides comprehensive assessment across all International Classification of Functioning, Disability and Health domains” [45, p. 619].
FOCUS^®^			✓	✓	2	“The constructs used in the FOCUS are derived from the ICF framework to measure changes in communication and their impact on participation. The response set in part II of the FOCUS (i.e., “cannot do at all” to “can always do without help”) was designed to evaluate the shift from capacity to performance by evaluating the level of assistance required to complete items successfully” [46, p. 51].
QYPP		✓	✓		2	“In developing the new instrument, we differentiated activities from participation at the level of ICF sub-domains, regarding activities as simpler elements of functioning at body level while participation usually includes those sub-domains made up of a number of activity functions and where the result is of intrinsic social and personal importance” [47, p. 501].
MEVU		✓			1	“This new measure is conceptually grounded within the Activity level domain of the International Classification of Functioning, Disability and Health as a measure of a single visual ability construct” [48, p. 1].
CAP-HAND			✓		1	“The conceptual frameworks underlying the development of the Children's Assessment of Participation with Hands are the ICF and the ICF-CY, in combination with additional participation definitions/attributes proposed by Coster and Khetani” [37, p. 1046]. ICF provided only an initial framework for the measure's development.
CAPE and PAC			✓		1	“The CAPE and PAC both focus on a subset of the ICF domains of participation and are based on two taxonomies, or classifications, of leisure and recreational participation” [39, p. 7].
ICF-CY Based Questionnaire	✓	✓	✓	✓	4	“The ICF-CY based questionnaire for children with autism comprised 4 domains: body functions, activities, participation and environment” [49, p. 679].
PEM-CY			✓	✓	2	“As defined by the International Classification of Functioning, Disability, and Health (ICF), participation and environment are multidimensional constructs that have been challenging to measure” [38, p. 238]. The ICF provided an initial framework for the measure's development.

## Discussion

This study is the first of which we are aware to identify ICF-based clinical measures for children with NDD. We have reported the psychometric properties and characteristics of 14 measures that are grounded in the ICF framework, using the information gathered from the initial development studies. We also identified the prominent ICF conceptual domain(s) that these measures represent, and the extent to which the framework was captured, including its interactive nature. There may be more ICF-based measures for this population that exist outside the clinical context (i.e., educational-based measures) and some of these tools may be applicable to other settings; however, the intent of this study was to examine how ICF-based clinical measures were operationalized in practice. Therefore, only health-focused databases were consulted.

The initial development studies for the selected measures included varying levels and types of psychometric properties conducted and described. Some studies [ICF-CS (41), GMFCS-ER (35), BFMF (43), MEVU (48), and PEM-CY (38)] placed emphasis on the process that the research team experienced when developing the measure, rather than describing specific psychometric characteristics of their measure. These studies had concurrent publications that described the conceptual processes and psychometric testing separately. The remaining studies combined psychometric testing with the measure's development process. The most common psychometric tests that were completed were interrater and test-retest reliability as well as content validity. It is important for clinical instruments to demonstrate good psychometric properties (27), and 64% of the measures were introduced with some form of psychometric testing conducted. These results provide a descriptive overview of the function-focused tools developed in the field of childhood NDD, but since potential subsequent psychometric studies were not included in this study, it is difficult to provide comment on the overall rigor of the state of function-focused tools in this field. Future research should examine levels of rigor found in the psychometric properties of the listed tools.

The 14 measures varied in their constructs of interest, age ranges, and diagnoses. These constructs ranged from very specific functional skills [i.e., BFMF: bimanual fine motor function (43)] to broader areas of interest [i.e., ICF-CY-Based Questionnaire: building a functional profile for children diagnosed with ASD (49)]. For age applicability, two measures [CAP-HAND (37) and CAPE & PAC (39)] had expanded upper-age ranges to 21 years old. Many measures focused on specific diagnoses: CP, ASD, and communication disorders. With the broad spectrum of diagnoses involved in DSM-5's definition of NDD, this highlights the need for great representation in other NDD populations. To fill these gaps, measures like the ICF-CS have been continually adapted with subsequent publications to explore the clinical utility of this measure in multiple communities within disability research and practice, including within NDD (50). These diagnosis-based populations include ASD, CP, and ADHD, but the outreach in these diagnosis populations continue to grow today (50, 51).

Furthermore, these results indicated that measures such as the CAP-HAND (37), CAPE & PAC (39), and PEM-CY (38) could be potentially used with any child or youth, regardless of whether they are diagnosed with any condition of NDD, as these measures are not diagnosis-specific. In addition, although a key population of the users of FOCUS^®^ (46) is young children with communication disorders, this tool is designed to address communication needs across all young children with or without disabilities. These findings are important, as they illustrate function-focused measures that examine abilities across diagnoses/conditions – an emerging trend (15). With the various diagnoses categorized within NDD, these measures have a wider scope in reaching different communities, thus creating more opportunities to utilize the concepts of the ICF in clinical and research settings. It is important to note that the ICF is still considered a contemporary framework, and that measures are continuing to be developed, such as the MEVU (48) that was published 1 month prior to conducting the database search for this narrative review.

With the heterogeneity of functional abilities within NDD, and the emerging measures that are being developed without a focus on any specific diagnosis, non-diagnostic ICF-based measures create opportunities for further examination of the continuum of abilities across diagnoses. By doing so, the goal of these measures shifts toward capturing profiles of individual abilities as well as unique differences among children (12). Furthermore, there is some evidence in today's literature that indicates that neurological similarities (i.e., brain structure/activity) that may affect an individual's social communication abilities may exist across diagnoses such as autism and attention deficit hyperactivity disorder; this shows that a child's overall abilities may also overlap across diagnoses (52). This example can be used to challenge the ways in which we can define, diagnose, and “treat” NDD, specifically with how we approach functional perspectives for these populations (53). Measurement tools may still involve neurophysiological processes in their design, but by focusing on a more individualistic foundation, this shift in thinking may better suit the cultural direction of how function-focused care is understood with today's ideas (12). By utilizing these measures across the populations of NDD, we better understand the diversity in the needs of children within their communities. These needs may exist beyond the core domains (i.e., addressing participation needs), and can potentially extend to how we can utilize these measures to improve the overall quality of life of children with NDD (13, 27, 28). As the ICF promotes this understanding that these four domains can build a unique functional profile of a child, the ICF can be seen as a supporting resource within the use of family-centered care to support a child's individualized needs. When this type of care continues to play prominent roles in the design and development of intervention plans for these individuals with disabilities, this may need to be more apparent in the tools that we develop as well. This approach utilizes the biopsychosocial model and will be a more relevant application of the ICF. Furthermore, with children receiving school supports that are often integrated with health services, exploring function-focused measures that are applicable in educational settings, such as the Functional Abilities Classification Tool (54), is also important to examine in future work.

The development studies demonstrated variability in how ICF concepts were foundational within their measures. Some studies explicitly stated that their measure was conceptually based in the ICF whereas others used the ICF to develop their measure's items or constructs. Both the *Participation and Activities* domains were predominately represented throughout all measures whereas *Contextual Factors* and *Body Structures and Function* were not as prominent. The ICF-CS (41) and ICF-CY-Based Questionnaire (49) utilized a holistic approach of the framework rather than focusing on specific domains, and this is evident simply looking at the naming of these tools. Other measures utilize the ICF combined with other frameworks, such as the CAP-HAND (37), that uses the definitions provided by both the framework and what is described by the authors of the PEM-CY (38) to configure a definition for participation that is suitable for the needs of CAP-HAND (37). These results align with the literature, specifically regarding the shift in thinking the ICF proposes: this framework has motivated health service providers to focus beyond “body structures and functions” to include the other roles (i.e., activities, participation) that can impact a child's level of functioning and health (55, 56).

There are different ways that tools and measures interact with the components of the ICF, and some measures can still utilize this framework without using it for its conceptual basis. It is here that the use of the ICF linking rules may become more relevant, as the rules provides an effective method to link meaningful “concepts” of non-ICF-based measures to the most precise category(s) in the ICF framework (57). These concepts could describe health condition, functional activities or any of the contextual factors (31, 57). This “linking process” differentiates from what is being studied in this narrative review, in that we are examining the extent to which ICF-based measures involve the domains of the framework, and the dynamic interactions they capture. This review focused on identifying measures that used the ICF for the initial conception, rather than the measures that have only considered the ICF post-publication or in an “after-the-fact” exploration. With the linking process, the developers of non-ICF based measures undergo the steps required in understanding the ICF to link certain items of their measure to the most relevant domains of the framework (58). However, the use of linking individual concepts of a measure to the ICF framework may not be as effective in demonstrating the interaction between the concepts, especially between activities/participation and contextual factors (59). Of the identified measures, 71% utilized more than one domain of the ICF, often highlighting the various ways in which the nuanced interactions influenced the development of the tool. For example, the PEM-CY (38) evaluates both participation and environmental factors in different settings, and can provide problem-solving strategies to adjust contextual factors within these settings to support further participation (38). As described earlier, the dynamic nature of the interaction of these non-linear domains is one of the most easily identifiable components of the framework. Although there is variability with how these interactions are explicitly described, when a measure is ICF-based and correctly utilizes the framework as a core component, the interactions of the domains are more likely to be inherently captured within the use of the measure.

## Limitations

There are a couple of limitations to report about this study. To begin, there were varying levels of psychometric data that emerged across the initial development studies of the selected measures. While psychometric testing of measures is an ongoing process, we recognize that the original development manuscripts would only have captured psychometric testing at its initial development, and that subsequent studies could have tested additional properties, potentially with other populations of children. We also recognize that the contributors of the development studies may have differed from the original developers of the measure. The aim of this review was to provide an overview of the current ICF-based measures developed for children with NDD, in which we focused on using the development studies as the main sources for this work.

A second limitation relates to study screening process. We selected measures that explicitly used the ICF in the screening of study abstracts in the identified health databases, either in the development study, or in a subsequent published study of the measure that was used to locate the original manuscript. Although there is the potential for other measures to incorporate the ICF framework in some capacity in the development and/or design of their measure, the focus of this work was to identify the measures that explicitly used the ICF and its domains as a foundational element in its work.

## Conclusion

This narrative review can serve as a potential resource for clinicians/researchers looking to use measures grounded in the ICF framework for children with NDD. These 14 measures can play important roles in creating effective applications of the ICF for exploring child functioning in both research and practice (15, 54, 60). As measures are continuing to be developed using the ICF framework at their foundation, this emerging knowledge can help inform function-focused care. By understanding how function-focused care is operationalized within the measures that we create, we are also able to better understand functioning in clinical care for children with NDD, and whether there are gaps in what is measured. These gaps are also evident in NDD populations where these ICF-based tools are not applied. Future research can explore the expansion of existing ICF-based measures across NDD populations and ages (i.e., adults), in addition to examining measures that impact functioning in other childhood contexts (e.g., home and educational settings).

## Author Contributions

KS completed the search and wrote the manuscript with support from BD, PR, and MZ. BD supervised the project, with help from PR and MZ as part of the supervising committee. PR, MZ, and BD conceived the original idea. All authors reviewed the results and contributed ideas to the final manuscript.

## Conflict of Interest

The authors declare that the research was conducted in the absence of any commercial or financial relationships that could be construed as a potential conflict of interest.

## Publisher's Note

All claims expressed in this article are solely those of the authors and do not necessarily represent those of their affiliated organizations, or those of the publisher, the editors and the reviewers. Any product that may be evaluated in this article, or claim that may be made by its manufacturer, is not guaranteed or endorsed by the publisher.
